# Fahr's Syndrome Misdiagnosed as Schizophrenia: A Case Report

**DOI:** 10.7759/cureus.1071

**Published:** 2017-03-02

**Authors:** Syeda Naqvi, Samiullah Arshad, Rida Hanif, Khaled Abdelmaqsoud Hamed Elfert

**Affiliations:** 1 Jinnah Postgraduate Medical Centre, Jinnah Sindh Medical University (SMC); 2 Internal Medicine, Combined Military Hospital Lahore, Pakistan; 3 Department of Psychiatry & Behavioral Sciences, Jinnah Postgraduate Medical Centre; 4 Internal Medicine, Hamad Medical Corporation

**Keywords:** fahr syndrome, adhd, schizophrenia, parathyroid, calcium, hallucination, bilateral calcification

## Abstract

Fahr's syndrome is a rare disease entity which presents with multiple neurological symptoms like movement disorder or cognitive impairment. We describe a case of a young male patient who presented with symptoms mimicking schizophrenia. He failed to improve despite medical management. He developed an episode of seizure which prompted us to make a computed tomography (CT) scan of the brain, revealing bilateral calcification of basal ganglia, despite normal serum calcium and parathyroid hormone (PTH) levels. This case experience explains the need to rule out all pathological causes of hallucinations before making a diagnosis of schizophrenia.

## Introduction

Fahr's syndrome is an infrequent disease and was first described in 1930 by a scientist named Karl Theodor Fahr. This disease is characterized by bilateral symmetrical calcification of basal ganglia. It can be idiopathic, genetic, or secondary to endocrine abnormalities [1]. Mostly familial, Fahr's syndrome is autosomal dominant and genetically heterogeneous [2]. Endocrine abnormalities include variations in calcium metabolism and parathyroid hormone (PTH) levels, hypothyroidism, thyroid adenoma, Graves' disease, hyponatremia, and phosphate alterations [3]. Clinical presentation includes both neurological and behavioral symptoms. Neurological symptoms include Parkinson’s disease-like movement disorder, vertigo, epilepsy, syncope, cerebellar ataxia, and dementia [4-5]. Psychiatric symptoms include paranoid ideation, delusions, low mood, lack of interest, and auditory and visual hallucinations [3, 6-7].

## Case presentation

A 21-year-old Asian male presented to the clinic with a history of increased paranoia, irrelevant talk, self-talk, and decreased sleep for the last two years. He has no family history of any psychiatric or medical illness. He had attained all his milestones on time, and he was an active student in his school. According to his parents, the patient's symptoms had started nine years ago when he had started to show inattentiveness, impulsivity, and hyperactivity. He was diagnosed with attention deficit hyperkinetic disorder (ADHD). His parents were counseled on behavioral therapy and the need for medications. His parents refused medications and agreed to behavioral therapy. They changed his environment. But he became more irritable and aggressive by the age of 16 years and his symptoms kept worsening.

**Figure 1:**
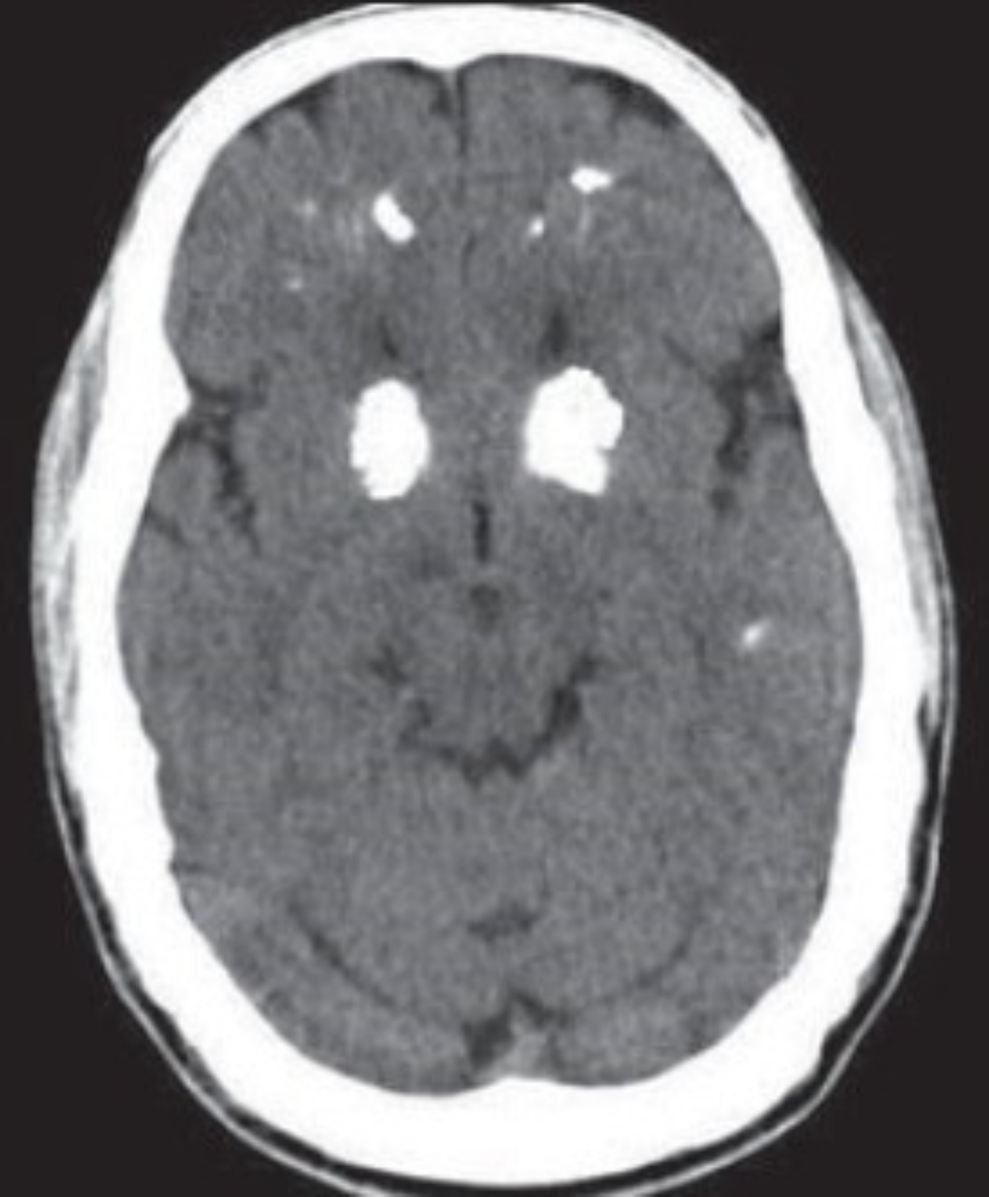
Bilateral calcification in basal ganglia

At the age of 18 years, he consulted a psychiatrist due to his symptoms of auditory and visual hallucinations, indifferent attitude, increased sexual desire, and aggressiveness. After excluding any illicit drug abuse and other causes, he was diagnosed with schizophrenia. He was given risperidone 2 mg and procyclidine hydrochloride 5 mg twice a day orally. He was regularly followed-up in the clinic. He remained symptom-free when he was taking his medications. After two months, he had an acute episode of seizure which lasted less than five minutes. On mental state examination, the patient was sitting comfortably, but his mood was anxious. He had decreased attention span and his thoughts and perceptions were distorted due to auditory and visual hallucinations. His judgment, abstract thinking, and memory were also distorted. According to his history, he had decreased appetite and sleep for the past two months since he had stopped taking his medication. He was observed in the hospital and a head computed tomography (CT) scan was ordered, which showed bilateral calcification in basal ganglia as can be seen in Figure 1.

Complete blood count, erythrocyte sedimentation rate (ESR), urinary analysis, basal metabolic panel, urinary copper level, total plasma parathyroid hormone (PTH) level, and electroencephalogram (EEG) got done. All his lab results were evaluated to be normal, including the urinary copper level of 71 mcg and ceruloplasmin levels of 0.28 mcg. Both sleep and wake EEG came back normal. Complete physical examination revealed no other significant findings. After ruling out any metabolic causes, electrolyte abnormalities, infections, and toxic or traumatic etiology, the patient was counseled for Fahr's syndrome. As there is no well-known treatment for Fahr's syndrome, the patient was managed with risperidone and procyclidine. The patient has been responding well to his medications for the last six months.

## Discussion

Presentation of Fahr's syndrome and its association has been extremely vague. Based on a literature review, it can be concluded that it is associated with different psychiatric illnesses like anorexia nervosa, mania, dementia, psychosis, and depression [8-9]. But symptoms like auditory and visual hallucinations are also linked to many diseases other than psychiatric illness. When we diagnose our patient with schizophrenia, we should always try to exclude drug abuse and other possible organic lesions in the brain which might cause these symptoms. We also need to divert our attention to such very rare causes like Fahr's syndrome [10]. The clinical criteria to diagnose Fahr's syndrome includes bilateral calcification on CT brain, autosomal dominant inheritance, the absence of any infection, toxin or drug, absence of mitochondrial dysfunction, and presence of progressive neurological dysfunction [1]. This case was quite unique in the sense that there is no family history of Fahr's syndrome and the patient was so young. For a long time, the patient only presented with psychiatric symptoms, no neurological symptoms were appreciated in the earlier course. So it is essential to keep it in mind other causes too when dealing with a patient with a combination of psychiatric and behavioral issues. 

## Conclusions

Clinical findings of Fahr's syndrome vary from neurological disorder to those mimicking schizophrenia. In this case, there were no neurological symptoms, and this patient only presented with psychiatric symptoms of auditory and visual hallucination accompanied by paranoid ideation. For a psychiatrist, it is essential to rule out organic brain disorders before labeling a patient, especially one who is young and has no family history of psychiatric illness.

From the literature review, it is significant that there is no case of Fahr's syndrome which manifests in such an early age with symptoms of ADHD and then ending up in visual and auditory hallucinations without any alteration in calcium levels.
